# The oxidative costs of reproduction are group-size dependent in a wild cooperative breeder

**DOI:** 10.1098/rspb.2015.2031

**Published:** 2015-11-22

**Authors:** Dominic L. Cram, Jonathan D. Blount, Andrew J. Young

**Affiliations:** Centre for Ecology and Conservation, College of Life and Environmental Sciences, University of Exeter, Penryn Campus, Cornwall TR10 9FE, UK

**Keywords:** oxidative stress, offspring care, antioxidants, cooperative breeding, costs of reproduction, life-history trade-offs

## Abstract

Life-history theory assumes that reproduction entails a cost, and research on cooperatively breeding societies suggests that the cooperative sharing of workloads can reduce this cost. However, the physiological mechanisms that underpin both the costs of reproduction and the benefits of cooperation remain poorly understood. It has been hypothesized that reproductive costs may arise in part from oxidative stress, as reproductive investment may elevate exposure to reactive oxygen species, compromising survival and future reproduction and accelerating senescence. However, experimental evidence of oxidative costs of reproduction in the wild remains scarce. Here, we use a clutch-removal experiment to investigate the oxidative costs of reproduction in a wild cooperatively breeding bird, the white-browed sparrow weaver, *Plocepasser mahali*. Our results reveal costs of reproduction that are dependent on group size: relative to individuals in groups whose eggs were experimentally removed, individuals in groups that raised offspring experienced an associated cost (elevated oxidative damage and reduced body mass), but only if they were in small groups containing fewer or no helpers. Furthermore, during nestling provisioning, individuals that provisioned at higher rates showed greater within-individual declines in body mass and antioxidant protection. Our results provide rare experimental evidence that reproduction can negatively impact both oxidative status and body mass in the wild, and suggest that these costs can be mitigated in cooperative societies by the presence of additional helpers. These findings have implications for our understanding of the energetic and oxidative costs of reproduction, and the benefits of cooperation in animal societies.

## 1. Introduction

Life-history theory assumes that reproduction entails a cost, and that investment in reproduction is therefore subject to trade-offs with other traits [1,2]. Indeed, there is extensive evidence that investment in reproduction can have a detrimental effect on future reproduction and survival [3–6]. Central to our understanding of these trade-offs, however, is the identification of the physiological mechanisms that underpin them [7].

Recently, oxidative stress has been highlighted as a potential physiological mediator of life-history trade-offs [8,9]. Oxidative stress occurs when reactive oxygen species (ROS) cause damage to proteins, lipids and DNA [10]. Exposure to oxidative stress is associated with increased rates of senescence, impaired future reproductive success and curtailed survival [11–13]. Under normal circumstances, the damaging effects of ROS are minimized by the body's complex antioxidant system [10]. However, during reproduction, oxidative stress may be promoted if the balance between ROS and antioxidants is disrupted, either by enhanced ROS generation, investment of antioxidants in reproduction rather than self-maintenance, or the combined effects of both [9]. As such, exposure to oxidative stress has been highlighted as a potential mechanism underpinning the costs of reproduction [8].

To-date, empirical studies investigating whether reproduction entails an oxidative cost have yielded equivocal results [14,15]. A recent meta-analysis highlighted some of the complexity that may contribute to this variation. While increased reproductive effort is positively associated with oxidative damage when the effect sizes of multiple studies are combined, such evidence may frequently be shrouded by pre-emptive ‘oxidative shielding’ tactics employed by breeders to mitigate such oxidative costs [14]. The elusive nature of empirical evidence for oxidative costs of reproduction may also be due in part to relevant studies being either correlative or conducted in captivity under artificial conditions. While correlative studies have revealed associations between reproductive effort and oxidative status, the causality of these links remains unclear, and they may instead reflect confounding differences in individual quality or terminal investment strategies [14]. Experimental manipulations of reproductive effort may therefore be necessary to reveal the oxidative costs of reproduction [16]. A number of valuable experimental studies have investigated links between reproduction and oxidative damage [14], yet these studies have been conducted almost exclusively in captive conditions (but see [17,18]). Studies in captivity typically feature ad libitum access to antioxidant-rich food, an absence of predation risk, and unnaturally low levels of competition, environmental stress and exercise. These relatively favourable conditions may relax the physiological demands on study animals, and thus diminish or even eliminate the trade-offs being investigated [8]. Favourable conditions in captivity may therefore explain why most laboratory studies have not found evidence of an oxidative cost of reproduction [14]. Advancing our understanding of the impacts of reproduction on oxidative status may therefore demand experimental studies, in natural populations living under ecologically realistic conditions [16,18].

If investment in reproduction does entail physiological costs, then evolution is expected to favour strategies that mitigate such costs. One such strategy may be helping behaviour in cooperatively breeding species [19,20]. In many cooperatively breeding societies, non-breeding helpers assist with the rearing of breeder's young, and in doing so may reduce the reproductive effort required of breeders (so-called ‘load-lightening’ [21,22]). Load-lightened breeders can enjoy improved reproductive success [23–25] and survival [26,27]. However, the physiological mechanisms that underpin these downstream benefits are unclear. If offspring care does entail an oxidative cost, then the lightening of individual workloads in cooperative groups may lead to concomitant reductions in the exposure of group members to oxidative stress, with those in larger groups conceivably enjoying ‘oxidative load-lightening’. Remarkably, the impact of helpers on the oxidative costs of reproduction in cooperatively breeding societies and the oxidative benefits of cooperation in group-living species remain largely unexplored [28,29].

Here, we use a clutch-removal experiment to investigate the impact of reproduction on oxidative status and body mass in a wild population of cooperatively breeding white-browed sparrow weavers, *Plocepasser mahali*. White-browed sparrow weavers live in year-round territorial groups of 2–12 birds throughout the semi-arid regions of sub-Saharan Africa [30,31]. Groups comprise a single dominant pair that completely monopolize within-group reproduction ([32]; though 12–18% of young are sired by extra-group males) and 0–10 subordinate males and females in approximately equal sex ratio [31]. This species shows well-developed cooperation, with most group members contributing to the care of young, sentinelling, territory defence and weaving [30,33]. Clutches of one to four eggs (mode: 2) are laid and incubated solely by the dominant female [32], while most group members contribute to the cooperative provisioning of nestlings and fledglings [31,33]. Breeders in larger groups (with more helpers) enjoy lower provisioning rates and higher annual rates of fledgling production [17,18]. Whether provisioning young entails costs in terms of impacts on either body mass or oxidative status, and whether such costs are mitigated by living in larger groups, has yet to be investigated.

Oxidative status is a complex, multi-faceted physiological state that can only be characterized by measuring multiple markers, including those indicative of antioxidant protection as well as oxidative damage [34]. We therefore investigate a suite of metrics of oxidative status, comprising a marker of oxidative damage and two markers of antioxidant protection. Lipids are a major target for ROS, and oxidative damage to lipids in cell membranes can be associated with cell death [35]. We measure plasma concentrations of malondialdehyde (MDA), a lipid peroxidation product. We also measure superoxide dismutase (SOD) activity in erythrocytes. SOD is a key intracellular antioxidant enzyme, forming part of the first line of defence against oxidative damage [36]. Finally, we measure the ability of a plasma sample to quench a free radical challenge *in vitro*, thus providing a functional measure of ‘total antioxidant capacity’ (TAC; and we statistically exclude the confounding effects of uric acid from this measure [37]). Owing to limited blood sample volumes, we did not measure other oxidative status markers. Furthermore, it has been highlighted that circulating oxidative status markers may not necessarily reflect oxidative status in other tissues [38]. As such, the suite of circulating markers used can only provide an estimate of the overall oxidative status of the organism.

Specifically, we use a clutch-removal experiment to investigate the costs associated with reproduction, by contrasting the plights of individuals in breeding groups whose clutches were experimentally removed at clutch completion (‘clutch-removal’ treatment) with those of individuals in breeding groups that were allowed to hatch and rear their clutches (‘control’ treatment). Individuals in both treatments were caught for the determination of their body mass and oxidative status, both at clutch completion and again one month later (when the control groups were provisioning their broods at their highest rates, and the clutch-removal groups were not breeding). First, we test whether reproduction entails a cost in terms of differential body mass reductions and deficits in oxidative status in the control treatment relative to the clutch-removal treatment, and whether such costs may be mitigated in larger social groups. We use a powerful repeated within-individual sampling approach, assessing all focal individuals for body mass and oxidative state metrics both before and after the treatment period. Second, we focus on the control breeding groups, to investigate whether higher rates of offspring provisioning *per se* are associated with larger body mass reductions and deficits in oxidative status during the peak provisioning period.

## 2. Material and methods

### (a) Study population

Data collection was conducted in the context of a long-term study, monitoring a population of white-browed sparrow weavers in an area of approximately 1.5 km^2^ in Tswalu Kalahari Reserve, South Africa (27°16′ S, 22°25′ E). All birds were fitted with a metal ring and three colour rings for identification; sex, dominance status and group size were identified using criteria detailed elsewhere [32,39,40]. Group size in our dataset ranged from two (the dominant pair with no helpers) to eight (the dominant pair with six helpers).

All captures, blood sampling and measurements were conducted by one person (SAFRING license 1444). Birds were captured individually at night, by flushing them from their roost into a custom capture bag. A blood sample (approx. 160 µl) was immediately collected from the brachial vein with a 26 g needle and heparinized capillary tubes. Blood was immediately separated by centrifugation (12 000*g* for 3 min, Haematospin 1400; Hawksley Medical and Laboratory Equipment, UK) and erythrocytes drawn from the cellular phase were lysed in the field (see the electronic supplementary material, S1). Body mass was recorded to the nearest 0.01 g (Durascale 100, MyWeigh, UK). Birds were then returned to their roosts to pass the remainder of the night.

### (b) Clutch-removal experiment

Nest searches were conducted every 1–2 days from November 2011 to April 2012. In this species, the dominant female lays one egg each morning on consecutive days until clutch completion. When eggs were discovered, the date of clutch completion could be determined by re-visiting the nest every afternoon until the same number of eggs was encountered on 2 consecutive days (‘clutch completion’). On the evening of the clutch completion day, the resident group members were captured, weighed and blood sampled. We aimed to capture all adult group members with the exception of the dominant female; dominant females were excluded from this study as catching them during incubation risks causing clutch abandonment. Groups were then randomly assigned to one of two treatments: the entire clutch of eggs was either collected from the nest (clutch-removal treatment; *n* = 9 groups), or handled and returned to the nest, allowing them to be incubated and reared as normal (control treatment; *n* = 11 groups). The subsequent breeding activity of all groups was then monitored, to confirm hatching dates for control groups and to confirm continued non-breeding status in the clutch-removal groups (no clutch-removal groups restarted breeding during the study period). All originally captured birds in both treatments were then captured again 25–35 days after clutch completion, when the nestlings in control groups were 10–12 days of age and therefore being provisioned at peak rates [33], and when clutch-removal groups were not breeding. At this point, all birds were weighed and blood sampled again, to investigate ‘final’ oxidative status and body mass. The two treatment groups did not differ in group size, clutch size, date of clutch completion, number of days between clutch completion and final captures, and number of birds captured at both time-points in each group (see the electronic supplementary material, table S2).

### (c) Provisioning observations

In the late incubation phase, all individuals except the dominant female were caught for the application of unique dye marks to their vent feathers. This allowed the identification of provisioning birds using video cameras placed beneath the nest. A tripod was placed at the nest 2 days before filming commenced, to allow the birds to habituate to its presence. On at least two mornings when nestlings were aged 9–12 days, video was recorded at the nest (186 ± 16 min starting at 06.52 h ± 16 min, mean ± s.d.). Individual feeding rates were calculated as nest visits per hour. Videos were recorded on mornings immediately preceding the collection of the final blood samples.

### (d) Oxidative status metric determinations

#### (i) Oxidative damage to lipids

Concentrations of MDA were determined in 10 µl plasma samples, using high-performance liquid chromatography (following [41]). A subset of plasma samples run in duplicate showed high repeatability (*F*_66,67_ = 15.92, *r* = 0.88, *p* < 0.001).

#### (ii) Enzymatic antioxidant protection

The SOD activity in erythrocyte lysate was determined using a colorimetric assay (Cayman Chemicals, USA) and a spectrophotometer (Spectramax M2; Molecular Devices, USA). Samples were diluted 1 : 200; 10 µl of diluted erythrocyte lysate was used for the assay. One unit is defined as the amount of enzyme needed to exhibit 50% dismutation of the superoxide radical; enzyme activities are reported as units per millilitre. SOD activities were highly repeatable between plates (*F*_37,38_ = 6.07, *r* = 0.72, *p* < 0.001).

#### (iii) Non-enzymatic antioxidant protection

We estimated non-enzymatic TAC by measuring the capacity of a plasma sample to quench a standardized free radical challenge. Plasma TAC was determined using a colorimetric assay kit (Cayman Chemicals, USA) and spectrophotometer (Spectramax M2; Molecular Devices, USA). Samples were diluted 1 : 10; 10 µl of diluted plasma was used for the assay. Plasma TAC values are expressed as Trolox-equivalent antioxidant concentrations (mM). TAC values were highly repeatable between plates (*F*_41,42_ = 8.20, *r* = 0.78, *p* < 0.001). To control for the potentially confounding effects of uric acid, we calculated residuals from a linear model with TAC as the response term and uric acid concentration as the sole predictor (following [37]). This yielded a measure of plasma antioxidant capacity excluding that arising from uric acid (hereafter termed ‘residual TAC’; see the electronic supplementary material, S3).

#### (iv) Uric acid

Plasma concentrations of uric acid were determined using a fluorescence assay kit (Cayman Chemical, USA) and spectrophotometer (Spectramax M2; Molecular Devices, USA). Samples were diluted 1 : 10; 10 µl of diluted plasma was used for the assay (see the electronic supplementary material, S4). Uric acid concentrations were highly repeatable between plates (*F*_39,40_ = 8.35, *r* = 0.79, *p* < 0.001).

### (e) Statistical analyses

Statistical analyses were carried out in R [42], using a stepwise model simplification approach [43]. Initially, all fixed terms of interest were fitted, followed by the stepwise removal of terms whose removal resulted in a non-significant change in deviance (using a likelihood-ratio test for model comparison), until the minimal adequate model (MAM) was obtained. Dropped terms were then added back in to the MAM to confirm their non-significance and were retained in the MAM if found to be significant in this context. The homoscedasticity and normality of residuals were inspected visually and where necessary response terms were transformed to satisfy these criteria. The significance of all terms was tested either by removing the terms from the MAM (if the term was in the MAM) or by adding the terms to the MAM (if the term was not in the MAM). Results are presented as means ± s.e., unless otherwise stated.

First, the effect of treatment on final measures of oxidative status and body mass was assessed. The final measure of a given metric was the response, and the level of that same metric at clutch completion was fitted as a predictor. This approach is statistically more powerful than modelling the effect of treatment on the change in a given metric, and can account for the effects of chance biases in the treatment groups in the initial levels of a given metric [43]. Where it did not significantly improve the fit of the model, the clutch completion predictor was removed during model simplification (though in each case we confirmed that its retention in the models did not qualitatively change the results). Treatment, group size and their two-way interaction were also fitted as predictors. Dominance/sex status was included as a three-level factorial predictor (dominant male, subordinate male and subordinate female), as the oxidative status of dominant individuals can be distinct to that of their subordinates, and this may impact behaviour and health [28,29,44]. Social group ID was fitted as the single random effect (while each individual had both clutch completion and final measures in the analysis, the former was a predictor and the latter a response in each case, and so the response contained no repeated measures of individuals).

Second, the effect of natural variation in individual provisioning rates on final measures of oxidative status and body mass was assessed (necessarily using data solely from the control groups). LMMs were fitted with an individual's provisioning rate (nest visits per hour), its dominance/sex status, social group size and brood size included as predictors, with social group ID fitted as the single random effect. Initially, we investigated whether the levels of a given oxidative status metric or body mass at clutch completion predicted the final (peak provisioning) levels of that same metric, using the subset of birds captured at both stages. However, the levels of each oxidative status metric at clutch completion did not significantly predict the final levels of that metric for the same bird (MDA (*n* = 22 birds), SOD (*n* = 21 birds) and residual TAC (*n* = 14 birds): all 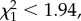 all *p* > 0.16), nor did they do so in the full dataset for both experimental treatments (see Results). For the analyses investigating the effect of provisioning rate on the final measures of each of the oxidative status metrics, the datasets were therefore expanded to include individuals sampled only at peak provisioning (to enhance the power of our analyses: MDA: *n* = 58 birds from 18 groups, SOD: *n* = 34 birds from 13 groups, residual TAC: *n* = 39 birds from 15 groups), and the levels of that metric at clutch completion were no longer fitted as a predictor. In contrast, as body mass at clutch completion was a strong positive predictor of final body mass at peak provisioning (see Results), the dataset for this analysis remained restricted to those birds captured at both clutch completion and peak provisioning.

## 3. Results

### (a) Does reproduction affect body mass and oxidative status?

#### (i) Body mass

**Figure 1.**
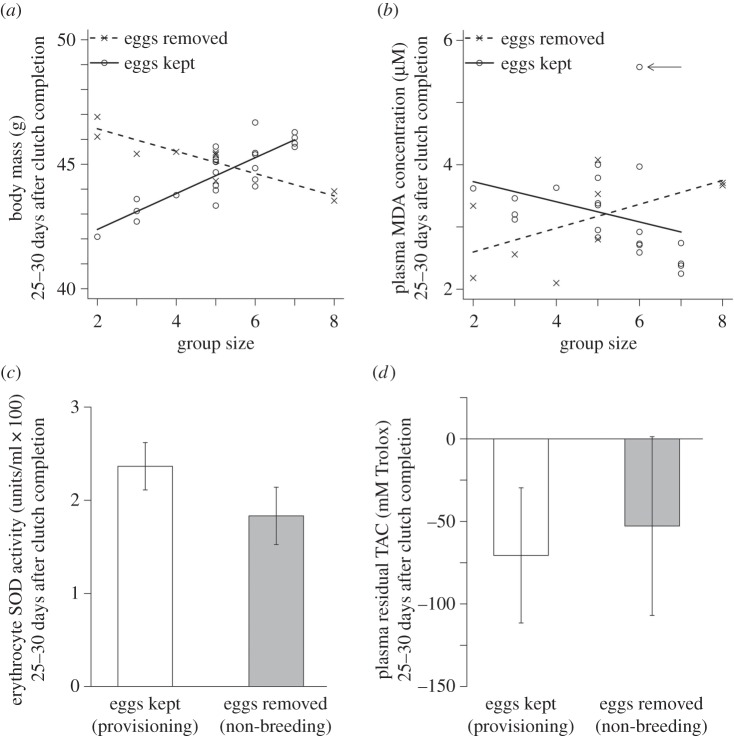
The effect of experimental clutch removal on (*a*) body mass (a significant interaction between treatment and group size), (*b*) plasma MDA concentration (a significant interaction between treatment and group size), (*c*) erythrocyte SOD activity (no significant effect of treatment), (*d*) plasma residual TAC (no significant effect of treatment). In (*a*,*b*), the lines represent linear mixed effect model predictions, and the points represent model residuals. In (*a*), the model contained clutch completion mass and the interaction between group size and treatment as predictors, and social group ID as the random effect. The predictions are for an individual of mean clutch completion body mass (44.75 g). In (*b*), the model contained the interaction between group size and treatment as the predictor, and social group ID as the random effect. The outlying high MDA value indicated by an arrow is not driving the interaction; its removal enhances the significance of the interaction (see results). In (*c*,*d*), bars represent the predicted means (± s.e.) for treatment in the minimal adequate model. In (*d*), the residuals are not distributed around zero as they were calculated using a dataset that also included the clutch completion TAC values, and the clutch completion residual TAC measures for all individuals are higher than their final residual TAC measures. Sample sizes, body mass: eggs removed (*n* = 10 birds from seven social groups), eggs kept (*n* = 24 birds from 11 social groups); MDA: eggs removed (*n* = 9 birds from seven social groups), eggs kept (*n* = 22 birds from 10 social groups); SOD: eggs removed (*n* = 10 birds from seven social groups), eggs kept (*n* = 21 birds from nine social groups) and residual TAC: eggs removed (*n* = 8 birds from six social groups), eggs kept (*n* = 14 birds from eight social groups).

An individual's final body mass (i.e. peak provisioning in control groups and when peak provisioning would have been in clutch-removal groups) was strongly positively predicted by its body mass 30 days earlier, at clutch completion (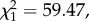 *p* < 0.001, *n* = 34 birds from 20 groups) and its dominance/sex status (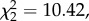 *p* = 0.005; dominant males: 46.44 ± 0.76 g, subordinate males: 45.23±1.33 g, subordinate females: 41.61±0.63 g). Controlling for these effects, there was also a significant interaction between experimental treatment and group size (figure 1*a*, 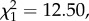 *p* < 0.001). The effect of treatment was strongest in smaller groups (groups of two to four birds), for which the final body masses of birds provisioning young (in control groups) were significantly lower than those not provisioning young (in clutch-removal groups; Welch two sample *t*-test: *t*_6.37_ = 3.67, *p* = 0.009). As our analysis controls for individual variation in body mass at *clutch competition*, this result reflects a differential within-individual decline in body mass in the control treatment, among members of small groups. In contrast, in larger groups (groups of five to eight birds), there was no significant effect of treatment (*t*_21.96_ = 0.34, *p* = 0.73); birds provisioning young had final body masses similar to those of birds whose clutch had been experimentally removed (figure 1*a*). The positive effect of group size on final body mass in the control treatment cannot be attributed to associated variation in brood size, as no correlation was found between brood size (one or two nestlings) and group size (*t*_20.12_ = 1.09, *p* = 0.29). There were also no associations between clutch completion body mass and group size or treatment (LMM with group ID as the random factor, both *χ*^2^ < 0.32, *p* > 0.57).

#### (ii) Plasma malondialdehyde concentration

As for the body mass findings above, final MDA concentrations were significantly predicted by the interaction between experimental treatment and group size (figure 1*b*, 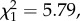 *p* = 0.016, *n* = 32 birds from 17 groups). In smaller groups (groups of 2–4 birds), control birds (who were provisioning nestlings) had significantly higher final MDA concentrations than clutch-removal birds (Welch two sample *t*-test: *t*_3.84_ = 2.85, *p* = 0.049). In larger groups (5–8 birds), there was no significant effect of treatment (*t*_17.42_ = 1.71, *p* = 0.10). As for body mass, the effect of group size on final MDA levels in the control treatment cannot be attributed to associated variation in brood size (see above). Final plasma MDA levels were also not influenced by treatment or group size as single terms (both 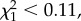 *p* > 0.74). The dataset contained a single outlying high final MDA value (indicated with an arrow in figure 1*b*), but this point was not driving the effect; its exclusion enhanced the interaction's significance (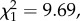 *p* = 0.002). An individual's final plasma MDA concentration was not significantly predicted either by its plasma MDA concentration at clutch completion or its dominance/sex status (both *χ*^2^ < 1.63, *p* > 0.44). There were no associations between clutch completion plasma MDA concentration and group size or treatment (LMM with group ID as the random factor, both *χ*^2^ < 0.10, *p* > 0.75).

#### (iii) Erythrocyte superoxide dismutase

Treatment did not significantly predict final SOD enzyme activity, either as a single term (figure 1*c*, 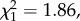 *p* = 0.17, *n* = 31 birds from 18 groups) or via an interaction with group size (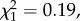 *p* = 0.66). SOD activity at clutch completion was a marginally non-significant positive predictor of final SOD activity (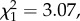 *p* = 0.08), but its retention or exclusion from the final model had no qualitative impact on the significance of treatment. Neither group size nor dominance/sex status significantly predicted final SOD activities (both *χ*^2^ < 2.12, *p* > 0.35).

#### (iv) Plasma residual total antioxidant capacity

Treatment did not significantly predict final plasma residual TAC, either as a single term (figure 1*d*, 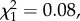 *p* = 0.78, *n* = 22 birds from 14 groups) or via an interaction with group size (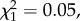 *p* = 0.82). Dominance/sex status significantly predicted final residual TAC (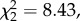 *p* = 0.015); subordinate females had lower residual TAC than both classes of male (dominant males: −60.00 ± 35.45 mM, subordinate males: 29.40 ± 66.43 mM, subordinate females: −216.93 ± 46.85 mM). Residual TAC at clutch completion did not significantly predict final residual TAC (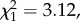 *p* = 0.078), although there was a weak trend towards consistency between the two time points. Neither blood sampling lag nor group size affected final residual TAC (both 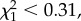 *p* > 0.58). Final plasma uric acid concentration was not significantly predicted by any of the model predictors (see the electronic supplementary material, S4).

### (b) Among provisioning birds, do those provisioning at a higher rate suffer greater deficits in body mass and oxidative status?

#### (i) Body mass

**Figure 2.**
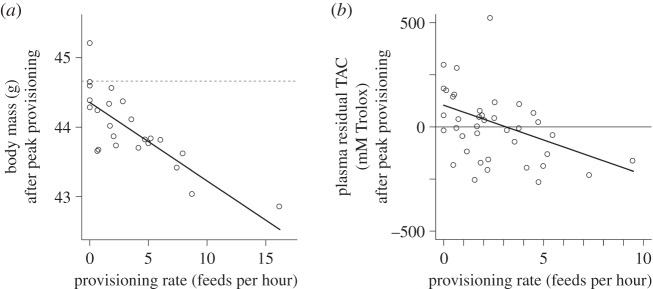
(*a*) The effect of natural variation in individuals' provisioning rates on their body mass at peak provisioning. Birds provisioning at a higher rate subsequently had a significantly lower body mass at peak provisioning, while controlling for the effects of individual variation in body mass at clutch completion and dominance/sex status (see Results). The solid line represents the prediction from a mixed effects model containing clutch completion mass and provisioning rate as predictors, and social group ID as the random effect. The predictions are for an individual of mean clutch completion body mass (44.66 g—indicated by the dotted line) and the points represent residuals from this model (*n* = 24 birds from 11 social groups). (*b*) The effect of natural variation in individuals' provisioning rates on their plasma residual TAC at peak provisioning. Birds provisioning at a higher rate subsequently had lower plasma residual TAC. The diagonal solid line represents the model predictions from a mixed effects model in which provisioning rate was the only predictor and social group ID the only random effect, and the points represent residuals from this model (*n* = 39 birds from 15 social groups).

An individual's body mass at peak provisioning was strongly positively predicted by its body mass 30 days earlier, at clutch completion (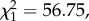 *p* < 0.001, *n* = 24 birds from 11 groups). Body mass at peak provisioning was also predicted by dominance/sex status (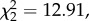 *p* < 0.001; dominant males: 46.44 ± 0.76 g, subordinate males: 45.23 ± 1.33 g, subordinate females: 41.61 ± 0.63 g), and was greater in larger social groups (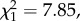 *p* = 0.005). Controlling for these effects, an individual's body mass at peak provisioning was significantly negatively predicted by its provisioning rate in the preceding days (figure 2*a*; 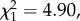 *p* = 0.027). Birds provisioning at higher rates subsequently had lower body masses. There was no additional effect on body mass at peak provisioning of variation in brood size (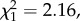 *p* = 0.14).

#### (ii) Oxidative status

Mirroring the relationship for body mass, an individual's plasma residual TAC at peak provisioning was significantly negatively predicted by its provisioning rate in the preceding days (figure 2*b*; 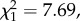 *p* = 0.006), after controlling for a significant effect of dominance/sex status (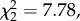 *p* = 0.02; dominant males: 14.79 ± 49.15 mM, subordinate males: 159.65 ± 47.76 mM, subordinate females: 36.50 ± 62.05 mM). Birds provisioning at higher rates subsequently had lower residual TAC measures. There was no additional effect on plasma residual TAC at peak provisioning of an individual's group size or the brood size that it was tending (both 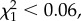 *p* > 0.81).

An individual's plasma MDA concentration at peak provisioning was not significantly predicted by its provisioning rate, brood size, dominance/sex status or group size (all *χ*^2^ < 0.70, all *p* > 0.40). Erythrocyte SOD activity at peak provisioning was not significantly predicted by provisioning rate, group size or dominance/sex status (all *χ*^2^ < 2.58, all *p* > 0.28, SOD activity was square-root transformed for normality of residuals). Brood size marginally significantly predicted final SOD activity (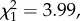 *p* = 0.046): SOD activities were higher in groups with two nestlings, compared with those with only one nestling.

## 4. Discussion

Our results provide rare experimental evidence of both a body mass and oxidative cost of raising young in a wild vertebrate, and suggest that the magnitude of these costs may be group-size dependent in this cooperatively breeding species. Compared to birds not caring for young following experimental egg removal, birds that reared young suffered a decline in body mass and elevated levels of a circulating marker of oxidative damage (MDA), but only in smaller social groups. Furthermore, our findings suggest that investment in nestling provisioning *per se* may contribute to the above costs: those birds that provisioned broods at higher rates lost the most body mass between clutch completion and peak provisioning and suffered greater reductions in antioxidant protection (residual TAC). Together, these results provide new evidence that reproduction can entail a twofold cost in the wild (impacting both body mass and oxidative status), and suggest that group-living may mitigate such costs in cooperatively breeding societies.

In small social groups (containing few or no helpers), birds provisioning offspring suffered greater body mass loss and higher levels of oxidative damage to lipids than birds whose eggs had been experimentally removed. While an energetic cost of reproduction is frequently documented (e.g. [45]), the evidence to-date for an oxidative stress cost of reproduction is equivocal (for reviews, see [14,15,46]). To the best of our knowledge, this is the first study to provide experimental evidence that reproduction increases exposure to oxidative damage in the wild [18]. Our findings therefore lend empirical support to the view that reproduction can entail an oxidative cost, and provide experimental support for the conclusion of a recent meta-analysis suggesting that there is a positive association between reproductive effort and oxidative damage [14]. As our focal individuals comprise dominant (reproductive) males as well as helpers of both sexes, all of whom provision the brood, these patterns likely reflect both an oxidative cost of reproductive effort *per se* in breeding males, and a novel oxidative cost of alloparental effort in helpers [29].

In this study, we assessed a circulating marker of oxidative damage to lipids, and thus cannot provide information about damage to other biomolecules (e.g. proteins or DNA). Our circulating markers may also not reflect variation in oxidative status in other tissues [38]. Nonetheless, circulating markers of oxidative status frequently correlate closely with components of health and survival (e.g. [11,39,47,48]), and our finding of elevated lipid damage in small breeding groups may therefore have important implications for future fitness.

While a recent meta-analysis found evidence that greater reproductive effort among breeders is associated with higher levels of oxidative damage, it also revealed an unexpected pattern: breeding individuals generally show *lower* levels of oxidative damage compared with those that are not breeding [14]. The authors suggest that lower levels of oxidative damage among breeders than non-breeders could reflect an adaptive strategy to provide ‘oxidative shielding’—decreasing exposure to oxidative stress during reproduction, in order to protect both adults and any developing offspring physiologically dependent on them (e.g. *in utero* or suckling offspring). Our apparently contrasting finding, that (in small groups) birds caring for young appear to show *higher* levels of oxidative damage than those whose clutches were removed, is not inconsistent with this ‘oxidative shielding hypothesis’, given the timing of our experimental manipulation. As both of our treatments used breeding groups that had produced complete clutches (in one treatment this clutch was then removed), individuals in both treatments may already have physiologically prepared to ‘shield’ against the oxidative challenge of reproduction [14], leaving the treatments conceivably contrasting more in their subsequent reproductive effort than in the extent of their pre-emptive shielding. Our findings lend support to the view that selection should favour protective ‘oxidative shielding’ mechanisms in order to reduce exposure to the oxidative stress that reproductive episodes could entail [14].

We find evidence for a body mass and oxidative damage cost of reproduction only in small social groups. This group-size-dependent treatment effect suggests that the costs of reproduction can be at least partially mitigated in larger groups, providing unique evidence, to our knowledge, of a potential oxidative benefit of group-living. Previous work on this species revealed no clear oxidative status or body mass benefit of living in a larger group during non-breeding periods [28], which is consistent with our experimental finding here: the benefit of larger groups in both currencies only becomes apparent during periods of reproductive effort. Such a benefit of group size during reproductive periods could arise because in this species, as for many other cooperatively breeding vertebrates, care for young is shared in larger social groups, reducing individual workloads and their associated costs [21,31,49,50]. Living in a larger group could also confer oxidative benefits through other mechanisms, such as improved foraging success resulting from reduced individual investment in vigilance [51]. Individuals in larger groups may also be of higher intrinsic quality or have access to superior foraging territories [52], leaving them better able to cope with the physiological challenges entailed in reproductive episodes.

The hypothesis that individuals in small social groups pay correspondingly larger costs specifically *because* they provision at higher rates is, however, lent support by our finding that birds that provisioned offspring at higher rates showed greater within-individual declines in body mass over the provisioning period and exhibited reduced levels of antioxidant protection at peak provisioning. While this result is correlative, it is consistent with the hypothesis that provisioning at higher rates demands greater energy expenditure and elevates generation of ROS [46], leading to the documented reduction in body mass and antioxidant defences [53]. These apparent costs of provisioning could also arise because frequent-provisioners experience a reduced *intake* of macro- and micro-nutrients, given the higher rates at which they donate food items to offspring [54]. It is perhaps surprising that the effect of reproduction on oxidative damage to lipids (MDA) revealed by our experiment was not reflected in our provisioning analyses as a positive association between provisioning rate and plasma levels of MDA. One possible explanation is that the variance in overall levels of metabolic work is greater in the contrast of breeding groups who raised their offspring and those whose eggs were experimentally removed, than among the provisioning birds in the correlative analysis (the vast majority of whom were provisioning young to some degree), thereby facilitating the detection of an effect on oxidative damage in the former approach. Alternatively, the oxidative damage costs that arise during the provisioning period may arise in part from changes in oxidative status that do not scale specifically with provisioning *rate*; they could, for example, arise in part from the time or effort expended when searching for the food items to be provisioned (which may vary with local conditions or the foraging skill of the provisioning bird). Finally, our measure of antioxidant protection may be correlated with another, unmeasured variable, which itself is associated with provisioning rate. Future work should manipulate provisioning rates or foraging efficiency to further clarify the associations between work rates, social group size and oxidative status in wild vertebrate societies.

Our study provides rare evidence that investment in reproduction entails a cost in terms of reduced body mass and elevated exposure to oxidative damage in a wild vertebrate. Uniquely, our results also suggest that helping behaviour in cooperatively breeding societies might entirely offset these costs in large social groups. Together, these findings have implications for our understanding of both the physiological costs of reproduction (which our results suggest may arise through both energetic and oxidative status mediated mechanisms) and the origins of helping behaviour in cooperatively breeding societies (which may have evolved to shield adults and developing offspring from these costs).

## Supplementary Material

ESM 1 blood sampling methods.docx

## Supplementary Material

ESM 2 association treatment table.docx

## Supplementary Material

ESM 3 residual tac 2.docx

## Supplementary Material

ESM 4 uric acid.docx

## Data accessibility

Data available from the Dryad Digital Repository: http://dx.doi.org/10.5061/dryad.j1305.
