# Supplementary material for: The oxidative costs of reproduction are group-size dependent in a wild cooperative breeder
Source: Proc Biol Sci. 2015 Nov 22;282(1819):20152031. doi: 10.1098/rspb.2015.2031 (PMC4685817; doi:10.1098/rspb.2015.2031)
Supplement: ESM 1 blood sampling methods.docx [file rspb20152031supp1.docx]

The oxidative costs of reproduction are group-size dependent in a wild cooperative breeder

Dominic L. Cram, Jonathan D. Blount & Andrew J. Young

**Electronic Supplementary Material S1: Blood processing methods**

After collection, blood was immediately separated by centrifugation in the field (12,000g for 3 minutes, Haematospin 1400; Hawksley Medical and Laboratory Equipment, UK). The time elapsed from sunset to capture was 272 ± 52 minutes (mean ± S.D.). The lag between bird capture and completion of blood sampling was minimized (mean ± S.D. = 213 ± 14 seconds).

Erythrocytes drawn from the cellular phase of the separated whole blood were immediately lysed. Lysis was standardized according to erythrocyte volume by combining them with four times their volume of ice-cold distilled water, mixing this solution, and placing it on ice for 5 minutes. This solution was then centrifuged for 3 minutes (12,000 × g) and the supernatant (erythrocyte lysate) drawn off. Plasma from the separated whole blood (for the determination of MDA, TAC and uric acid levels) and lysed erythrocytes (for the determination of SOD activities) were stored on ice until they could be transferred to liquid nitrogen on return to base camp (mean ± S.D. time from sampling to storage on liquid nitrogen: 110 ± 46 minutes). Samples were transported from the field site to the UK on dry ice where they were stored at -80°C until analysis within 12 months.
