# Supplementary material for: The oxidative costs of reproduction are group-size dependent in a wild cooperative breeder
Source: Proc Biol Sci. 2015 Nov 22;282(1819):20152031. doi: 10.1098/rspb.2015.2031 (PMC4685817; doi:10.1098/rspb.2015.2031)
Supplement: ESM 2 association treatment table.docx [file rspb20152031supp2.docx]

The oxidative costs of reproduction are group-size dependent in a wild cooperative breeder

Dominic L. Cram, Jonathan D. Blount & Andrew J. Young

**Electronic Supplementary Table S2: Treatment group associations**

| **Parameter** | **Treatment** | **Range** | **Mean** | **S.E.** | **Welch 2 sample t-test** |
| --- | --- | --- | --- | --- | --- |
| Group size | Control | 2-7 | 5.2 | 0.28 | t_12.4_= 0.70  p = 0.50 |
|  | Clutch removal | 2-8 | 4.7 | 0.67 |  |
|  |  |  |  |  |  |
| Clutch size | Control | 1-3 | 1.88 | 0.09 | t_23_ = 1.37  p = 0.19 |
|  | Clutch removal | 2 | 2 | 0 |  |
|  |  |  |  |  |  |
| Clutch completion date | Control | 17/12/11 - 10/3/12 | 23/1/12 | 6.9 | t_39.5_ = 0.30  p = 0.77 |
|  | Clutch removal | 8/1/12 -29/2/12 | 25/1/12 | 4.49 |  |
|  |  |  |  |  |  |
| Days from *clutch completion* to *final* blood samples | Control | 25-27 | 26.26 | 0.15 | t_33.5_ = 1.34  p = 0.19 |
|  | Clutch removal | 25-35 | 27.71 | 0.6 |  |
|  |  |  |  |  |  |
| Birds captured twice per group | Control | 1-4 | 2.18 | 0.38 | t_14.6_ = 1.76 |
|  | Clutch removal | 1-2 | 1.43 | 0.2 | p = 0.10 |

Supplementary table S1. Treatment group associations

Treatment groups did not significantly differ in group size, clutch size, date of clutch completion, the number of days between *clutch completion* and *final* blood sample collection, or the number of birds in each group captured at both *clutch completion* and *final* blood sample collection.
