# Supplementary material for: The oxidative costs of reproduction are group-size dependent in a wild cooperative breeder
Source: Proc Biol Sci. 2015 Nov 22;282(1819):20152031. doi: 10.1098/rspb.2015.2031 (PMC4685817; doi:10.1098/rspb.2015.2031)
Supplement: ESM 3 residual tac 2.docx [file rspb20152031supp3.docx]

The oxidative costs of reproduction are group-size dependent in a wild cooperative breeder

Dominic L. Cram, Jonathan D. Blount & Andrew J. Young

**Electronic Supplementary Material S3: residual TAC background and methods**

It has recently been highlighted that, in some avian species, a significant portion of the antioxidant activity measured by the TAC assay may be due to the antioxidant effects of uric acid, the primary nitrogen waste product in birds [1]. The importance of uric acid as an antioxidant *in vivo* remains unclear [2, 3], leaving avian plasma TAC values potentially confounded by the ‘incidental’ antioxidant activity of uric acid [4].

Indeed, in our plasma samples there was a strong linear relationship between uric acid concentration and TAC (see supplementary figure 1 below; linear mixed-effects model (LMM) with bird identity as the random factor: χ^2^_1_ = 56.43, p < 0.001, n = 50 samples; model estimate: 0.27 ± 0.064, conditional R^2^ = 0.40, see [5]). The model fit was not significantly improved by the addition of the quadratic polynomial of uric acid (χ^2^_1_ = 0.027, p = 0.87), and the standard linear-modelling diagnostic plots (for heteroskedasticity and normality of the sample and model residuals) were consistent with a linear relationship.

We therefore calculated residuals from a linear model with TAC as the response term and uric acid concentration as the sole predictor, to yield a measure of plasma antioxidant capacity excluding that arising from uric acid (hereafter termed ‘residual TAC’; see [4]).


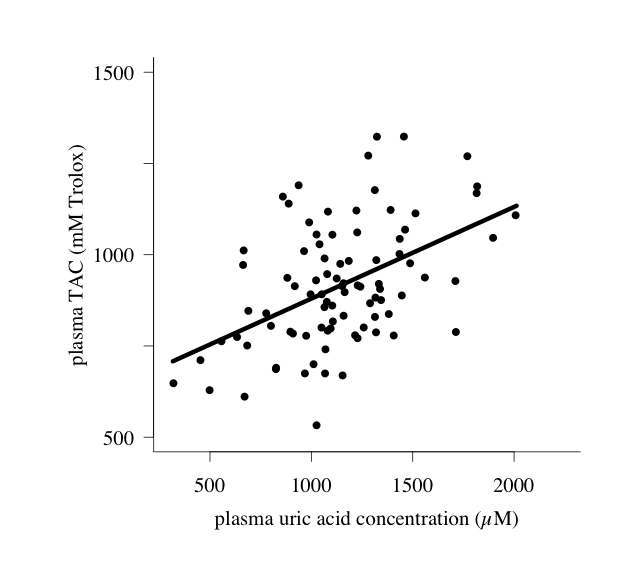


Supplementary figure 1: the linear correlation between plasma uric acid concentration and plasma TAC. The points indicate raw data, and the line indicates the regression line from a linear model with TAC as the response and uric acid as the only predictor.

While taking this ‘residual TAC’ approach avoids the confounding effects of uric acid [6], residual TAC still likely reflects only a portion of overall antioxidant protection (e.g. it does not comprise enzymatic antioxidant defences), and should therefore be interpreted with caution. Nonetheless, residual TAC has been used widely in ecological studies of antioxidant protection, and extensive evidence suggests it is a meaningful measure of circulating antioxidant activity, exhibiting associations with other antioxidants in serum [4, 6] and parameters related to health and fitness (e.g. growth rate, BMI, reproduction, parasite infection, sexual ornamentation [2, 3, 6-10]).

References

1. Robin J.-P., Cherel Y., Girard H., Géloen A., Le Maho Y. 1987 Uric acid and urea in relation to protein catabolism in long-term fasting geese. *J Comp Physiol [B]* **157**(4), 491-499. (doi:10.1007/bf00691834).

2. Cohen A.A., McGraw K.J. 2009 No simple measures for antioxidant status in birds: complexity in inter‐and intraspecific correlations among circulating antioxidant types. *Funct Ecol* **23**(2), 310-320. (doi:10.1111/j.1365-2435.2009.01540.x).

3. Kilgas P., Tilgar V., Külavee R., Saks L., Hõrak P., Mänd R. 2010 Antioxidant protection, immune function and growth of nestling great tits *Parus major* in relation to within-brood hierarchy. *Comp Biochem Physiol B* **157**(3), 288-293. (doi:10.1016/j.cbpb.2010.07.002).

4. Cohen A., Klasing K., Ricklefs R. 2007 Measuring circulating antioxidants in wild birds. *Comp Biochem Physiol B* **147**(1), 110-121. (doi:10.1016/j.cbpb.2006.12.015).

5. Nakagawa S., Schielzeth H. 2013 A general and simple method for obtaining R^2^ from generalized linear mixed-effects models. *Methods Ecol Evol* **4**(2), 133-142. (doi:10.1111/j.2041-210x.2012.00261.x).

6. Cohen A.A., Mauck R.A., Wheelwright N.T., Huntington C.E., McGraw K.J. 2009 Complexity in relationships between antioxidants and individual life-history parameters in a seabird and a songbird. *Oikos* **118**(12), 1854-1861. (doi:10.1111/j.1600-0706.2009.17785.x).

7. Alan R.R., McWilliams S.R. 2013 Oxidative stress, circulating antioxidants, and dietary preferences in songbirds. *Comp Biochem Physiol B* **164**(3), 185-193. (doi:10.1016/j.cbpb.2012.12.005).

8. Cohen A., Hau M., Wikelski M. 2008 Stress, metabolism, and antioxidants in two wild passerine bird species. *Physiol Biochem Zool* **81**(4), 463-472.

9. López-Arrabé J., Cantarero A., Pérez-Rodríguez L., Palma A., Alonso-Alvarez C., González-Braojos S., Moreno J. 2015 Nest-dwelling ectoparasites reduce antioxidant defences in females and nestlings of a passerine: a field experiment. *Oecologia*, 1-13. (doi:10.1007/s00442-015-3321-7).

10. López-Arrabé J., Cantarero A., Pérez-Rodríguez L., Palma A., Moreno J. 2014 Plumage ornaments and reproductive investment in relation to oxidative status in the Iberian Pied Flycatcher (*Ficedula hypoleuca iberiae*). *Canadian Journal of Zoology* **92**(12), 1019-1027. (doi:10.1139/cjz-2014-0199).
