# Supplementary material for: The oxidative costs of reproduction are group-size dependent in a wild cooperative breeder
Source: Proc Biol Sci. 2015 Nov 22;282(1819):20152031. doi: 10.1098/rspb.2015.2031 (PMC4685817; doi:10.1098/rspb.2015.2031)
Supplement: ESM 4 uric acid.docx [file rspb20152031supp4.docx]

The oxidative costs of reproduction are group-size dependent in a wild cooperative breeder

Dominic L. Cram, Jonathan D. Blount & Andrew J. Young

**Electronic Supplementary Material S4: uric acid methods and results**

*Methods*

Plasma concentrations of uric acid were determined using a fluorescence assay kit (Cayman Chemical, USA) and spectrophotometer (Spectramax M2; Molecular Devices, USA). Briefly, uricase catalyzes the conversion of uric acid in the sample to allantoin, hydrogen peroxide (H2O2), and carbon dioxide. Peroxidase subsequently catalyzes the stoichiometric reaction of H2O2, with ADHP (10-acetyl-3,7,- dihydroxyphenoxazine), producing the highly fluorescent compound resorufin. Resorufin fluorescence was measured with an excitation wavelength of 535 nm and an emission wavelength of 590nm. Samples were diluted 1:10; 10μl of diluted plasma was used for the assay. A subset of plasma samples were run in duplicate on separate plates, which confirmed that uric acid concentrations were highly repeatable between plates (F_39,40_ = 8.35, r = 0.79, p < 0.001).

*Results*

*Final* plasma uric acid concentration was not significantly predicted by treatment or group size (either as single terms or in an interaction), or dominance/sex status, blood sampling lag or plasma uric acid levels at *clutch completion* (all χ^2^ < 2.36, p > 0.12, n = 22 birds from 14 groups). There were no associations between *clutch completion* plasma uric acid levels and body mass or treatment (both χ^2^ < 0.18, p > 0.67), and a non-significant trend towards lower *clutch completion* plasma uric acid levels in larger groups (χ^2^_1_ = 3.10, p = 0.078).
